# Ketogenic Diet as an Epigenetic Therapy in *SETD1B*‐Related Epilepsy

**DOI:** 10.1002/acn3.70345

**Published:** 2026-02-19

**Authors:** Erica Tsang, Brian S. Gloss, Jessica P. Hayes, Andrew J. A. Holland, Manoj P. Menezes, Joceline A. Branson, Shekeeb S. Mohammad, Jingya J. Yan, Shrujna Patel, Velda X. Han, Russell C. Dale

**Affiliations:** ^1^ Kids Neuroscience Centre, the Children's Hospital at Westmead, Faculty of Medicine and Health University of Sydney Sydney New South Wales Australia; ^2^ The Children's Hospital at Westmead Clinical School, Faculty of Medicine and Health University of Sydney Sydney New South Wales Australia; ^3^ Westmead Research Hub Westmead Institute for Medical Research Westmead New South Wales Australia; ^4^ Douglas Cohen Department of Paediatric Surgery, the Children's Hospital at Westmead Clinical School, the Faculty of Medicine and Health The University of Sydney Sydney New South Wales Australia; ^5^ TY Nelson Department of Neurology and Neurosurgery, the Children's Hospital at Westmead The University of Sydney Sydney New South Wales Australia; ^6^ General Paediatrics Department Canterbury Hospital, Sydney Local Health District Sydney New South Wales Australia; ^7^ School of Mathematical and Physical Sciences, Faculty of Science University of Technology Sydney New South Wales Australia; ^8^ The Brain and Mind Centre The University of Sydney New South Wales Australia; ^9^ Khoo Teck Puat‐National University Children's Medical Institute, National University Health System Singapore Singapore; ^10^ Department of Paediatrics, Yong Loo Lin School of Medicine National University of Singapore Singapore Singapore

**Keywords:** butyrate, chromatin, neurology, precision nutrition, single cell rna sequencing

## Abstract

Histone lysine methyltransferases such as *SETD1B* regulate chromatin structure and gene transcription. Ketone bodies, including butyrate, act as histone deacetylase inhibitors. We report a 4‐year‐old boy with *SETD1B*‐related absence epilepsy, refractory to conventional medications, who achieved sustained > 90% seizure reduction on the Modified Atkins ketogenic diet. Single‐cell RNA sequencing of 25,159 peripheral mononuclear cells across 3 samples: baseline, 3 months on‐diet and age‐matched control, revealed widespread dysregulation of the patient's chromatin, ribosomal, immune and mitochondrial pathways at baseline, which were reversed with ketogenic therapy. These findings suggest that the ketogenic diet can improve gene regulation in chromatin‐mediated brain disorders.

## 1 Introduction

Mendelian Disorders of the Epigenetic Machinery (MDEMs) are monogenic conditions caused by pathogenic variants in chromatin‐modifying genes, resulting in transcriptional dysregulation and neurodevelopmental impairment. Variants in *SETD1B* (also named *KMT2G*) which encodes histone H3 lysine‐4 methyltransferase (H3K4), a catalytic subunit of the COMPASS complex, are associated with developmental delay, intellectual disability, autism spectrum traits and epilepsy, most commonly absence seizures [1]. SETD1B deposits H3K4me3 marks at CpG‐rich promoters to enable transcriptional activation and maintain promoter stability [2]. Pathogenic loss‐of‐function variants lead to altered promoter chromatin states, secondary DNA hypermethylation and global transcriptional dysregulation of neuronal gene networks [3, 4]. Here, we report a young boy with *SETD1B*‐related absence epilepsy which was refractory to conventional therapy and used single‐cell RNA sequencing (scRNA‐Seq) to examine the effects of the ketogenic diet (KD) on gene regulation.

## 2 Methods

### 2.1 Patient and Control

A 4.5‐year‐old male with de novo *SETD1B*‐associated refractory absence epilepsy referred to ketogenic service was recruited, and peripheral mononuclear cells were taken at baseline and 3 months on‐diet. A 5‐year‐old male with normal development and no medical history was recruited as an age and sex‐matched control. The control was on a standard Western diet (54% total energy intake as carbohydrate, 28% as fat, 18% as protein), providing a reference dietary composition that was distinct from the KD.

### 2.2 Modified Atkins Ketogenic Diet Treatment

Carbohydrate was initially restricted to 15 g/day, with fat provision at 65%–70% of total energy intake, and protein to appetite [5]. Despite initial food refusal associated with elevated blood ketones at 6.8 mmol/L, the patient became seizure‐free during the first week of diet initiation. A low glycemic index (GI) diet was adopted with gradual transition to MAD for safe ketosis. After 3 months on the MAD, the macronutrient composition of diet was 10 g of carbohydrate per day (2% total energy intake—EI), with 140 g fat (75% EI) and 96 g protein (23% EI), which was maintained thereafter. Twice‐daily blood ketone monitoring (Figure S1) indicated levels within ideal range (2–5 mmol/L) and good dietary adherence.

### 2.3 Single‐Cell RNA Sequencing and Bioinformatics

To characterise the transcriptomic changes in *SETD1B*‐related epilepsy following KD, scRNA‐Seq using whole leukocytes and HIVE single‐cell system was performed in pre‐diet, post‐diet and in the control (Data S1). ScRNA‐Seq data was analysed with *Seurat* R package (v5.3.1). Differentially expressed genes (DEGs) were defined at a false discovery rate (FDR) < 0.05 and subjected to Gene Ontology (GO) over‐representation analysis (ORA) using *clusterProfiler* (v4.19.1).

## 3 Results

### 3.1 Case History

A 4.5‐year‐old male with no significant family history was born at full term. From 2.5 years, he developed up to 30 atypical absence seizures per day, characterised by behavioural arrest, eyelid fluttering, upward eye deviation, with occasional head drops, unsteadiness and falls. Initial development was normal, and electroencephalogram (EEG) showed 3 Hz spike‐and‐wave discharges consistent with childhood absence epilepsy.

At 3.5 years, treatment with ethosuximide and sodium valproate was ineffective. From age 4, he experienced generalised tonic–clonic seizures (GTCS) lasting up to 6 min, generally associated with infections. Developmental concerns also emerged, including delays in gross and fine motor skills, speech, attention and concentration. Video EEG at age 4 captured 40 seizures in 24 h characterised by behavioural arrest, head drops and unsteadiness. The EEG again showed 3 Hz spike and polyspike.

From 4.5 years, he had multiple daily tonic seizures upon waking, characterised by eyelid fluttering, stiffness and unresponsiveness. Atypical absence episodes increased up to 50 per day. Lamotrigine resulted in cessation of GTCS, but further trials of levetiracetam, clobazam and phenobarbitone were either ineffective or poorly tolerated due to behavioural side effects. Despite treatment with ethosuximide, valproate, lamotrigine and clobazam, the absence seizures persisted.

Trio exome sequencing identified a de novo missense variant in *SETD1B* (c.5686A>G; p.Lys1896Glu), absent in gnomAD and not previously reported. The variant lies in a region highly intolerant to variation and was classified as likely pathogenic (American College of Medical Genetics and Genomics, ACMG, Class 4).

During the 3‐month trial of the diet, all other antiseizure medications remained unchanged. Within the first month on diet, absence seizures reduced from ~50 per day to 0–3 per day (> 90% reduction), with no further GTCS and associated falls. Seizure threshold when unwell and post‐ictal period appeared improved, with ~10 seizures/day during illness compared to > 50 per day.

Notably, improvements were observed in behaviour, language, memory (delayed story recall) and concentration (puzzle completion), with support of speech and developmental therapies. Epilepsy‐specific quality of life (QoL), measured using the PedsQL Epilepsy Module (0–100; higher scores indicate better QoL), improved from 50 at baseline to 61 after 3 months on KD. The Clinical Global Impression (CGI) score concurrently improved from 7 (severely ill) to 2 (much improved).

The patient has continued the MAD supplemented with medium‐chain triglyceride (MCT) oil for 1.5 years, maintaining a > 90% reduction in seizures compared to baseline. With optimisation of blood ketones at 4–5 mmol/L, absence seizures remain controlled at 0–3 per day. Since diet commencement (18 months) only one GTCS has occurred, associated with air travel and resolved without midazolam.

### 3.2 Cell Type‐Specific Gene Expression at Baseline

**FIGURE 1:**
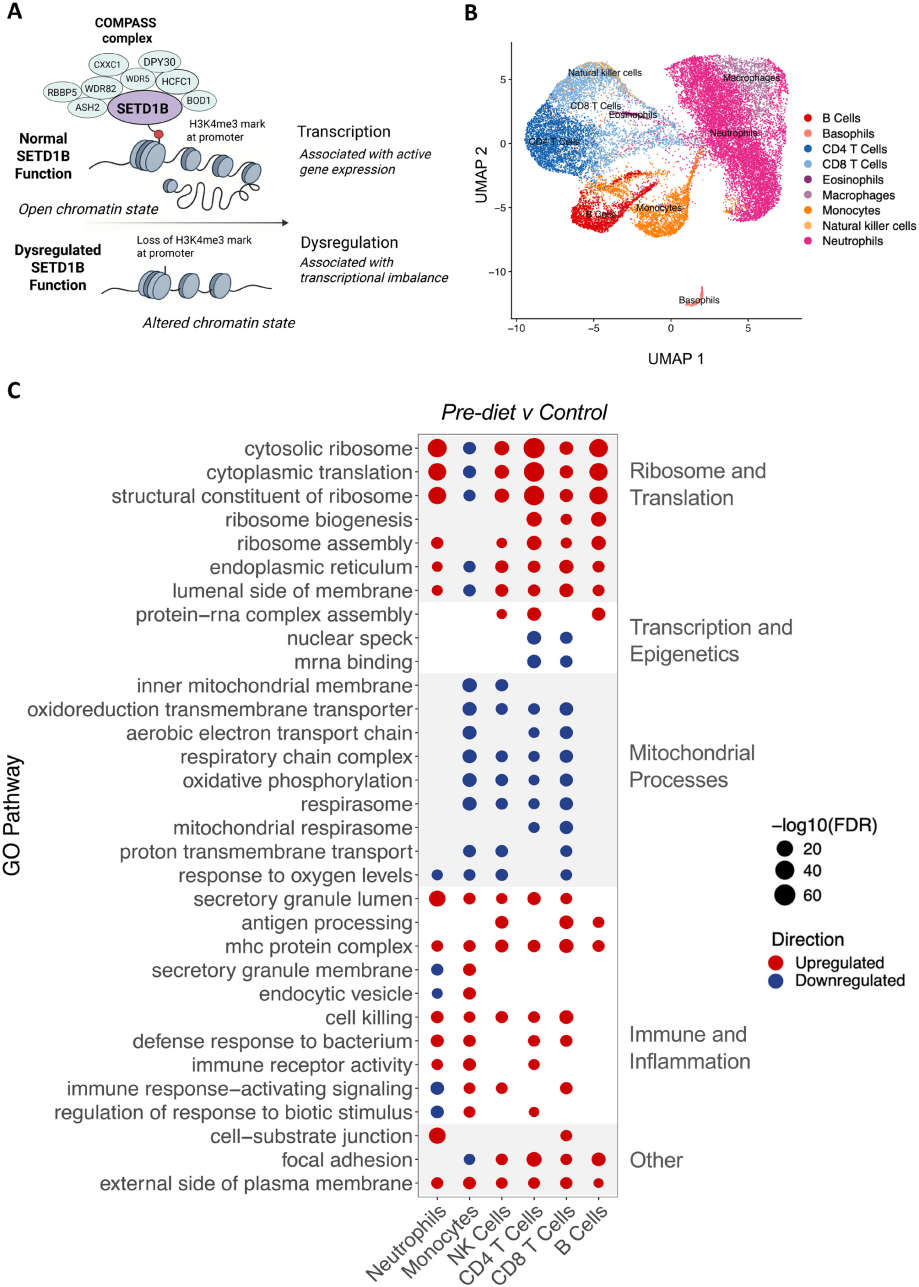
(A) Schematic of SETD1B function and transcriptional dysregulation in *SETD1B*‐related epilepsy. SETD1B forms part of the COMPASS complex that catalyses trimethylation of histone H3 at lysine 4 (H3K4me3) at gene promoters. Under normal conditions, H3K4me3 is associated with an open chromatin state and active transcription. Loss‐of‐function variants in *SETD1B* disrupt COMPASS activity, leading to reduced H3K4me3 deposition, chromatin compaction and transcriptional repression at key regulatory loci. This results in a global transcriptional dysregulation, contributing to the molecular pathology observed in *SETD1B*‐related epilepsy. (B) Uniform manifold approximation and projection (UMAP) of 3 samples (control, patient pre‐diet and patient post‐diet) showing 9 distinct cell types: Basophils, B cells, CD4^+^ T cells, CD8^+^ T cells, Eosinophils, Monocytes, Natural Killer cells, Neutrophils. (C) Dot plot showing the top 10 upregulated and downregulated Gene Ontology (GO) pathways for each cell type in the Pre‐diet versus Control comparison. These were then restricted to pathways shared across 2 or more cell types to highlight common biological processes. Ribosomal, translational and immune‐related pathways were predominantly upregulated, whereas epigenetic and mitochondrial processes were largely downregulated. Upregulated pathways are shown in red, and downregulated pathways in blue, with dot size indicating the statistical significance (−log_10_FDR).

Figure 1A illustrates the role of SETD1B in depositing H3K4me3 marks at gene promoters to encourage transcriptional activation, with loss or dysfunction of SETD1B leading to global transcriptional dysregulation. To explore these molecular effects, we performed scRNA‐Seq on a total of 25,159 cells across 3 samples (pre‐diet, post‐diet, control). Using uniform manifold approximation and projection (UMAP) analysis, 9 distinct cell types were identified (Figure 1B). Over‐representation analysis of significant DEGs identified the top 10 significantly upregulated and downregulated GO pathways for each cell type in Pre‐diet versus Control (FDR < 0.05) (Figure 1C). This revealed predominant upregulation of ribosomal/translational and immune/inflammatory pathways in *SETD1B* Pre‐diet versus Control, with concurrent downregulation of mitochondrial and transcriptional/epigenetic processes.

### 3.3 Effect of Ketogenic Diet on Cell Type‐Specific Gene Expression

**FIGURE 2:**
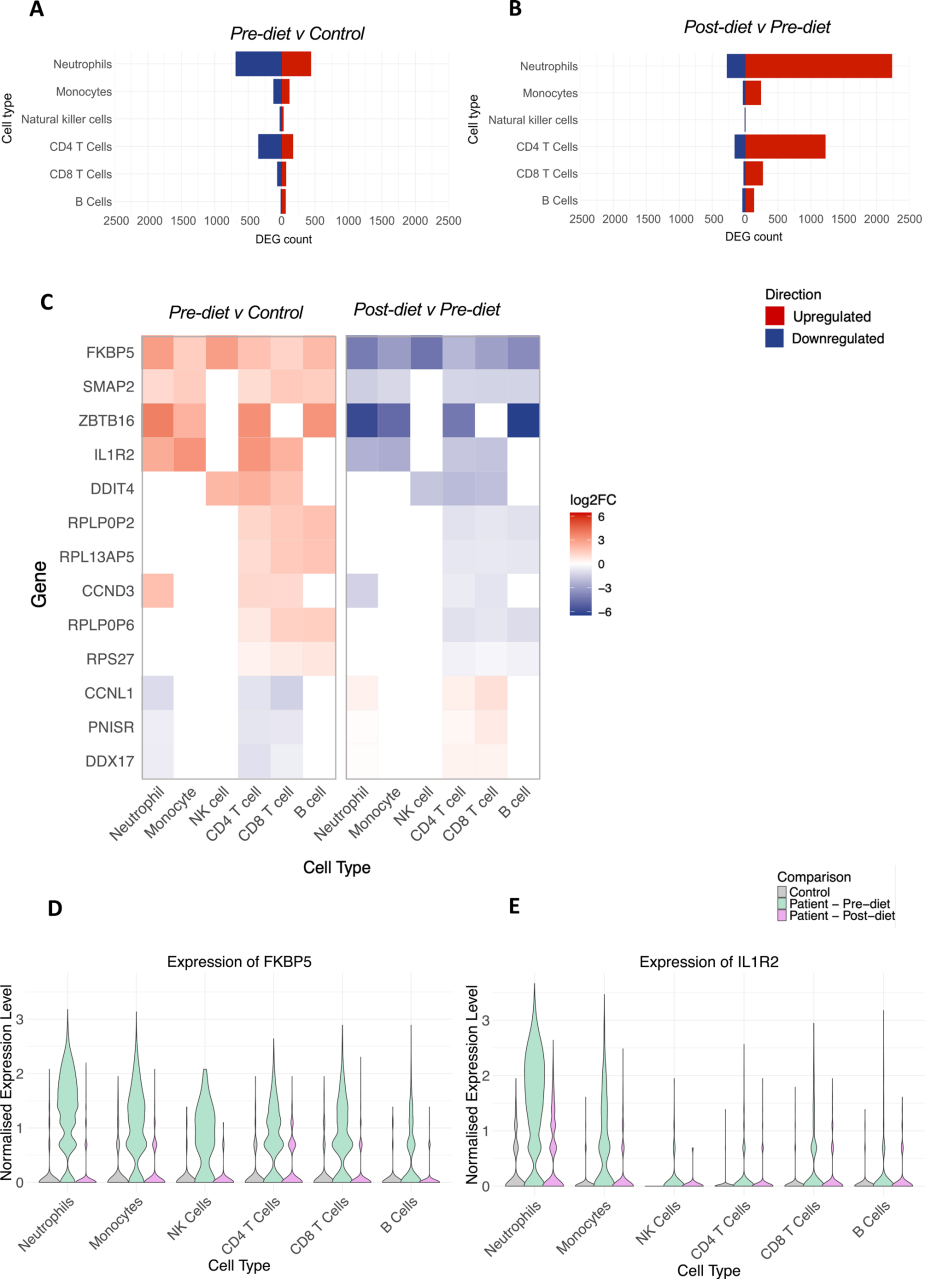
(A) Divergent bar plot showing differentially expressed genes (DEGs) across six cell types in the Pre‐diet versus Control comparison. Upregulated genes are shown in red and downregulated genes in blue. DEG counts ranged from 59 to 1129 across cell types, with a predominance of downregulated (blue) genes in neutrophils and CD4+ T cells. (B) Divergent bar plot showing DEGs across the same cell types in the Post‐diet versus Pre‐diet comparison. DEG counts ranged from 13 to 2510, with a predominance of upregulated (red) genes in neutrophils and CD4+ T cells. (C) Heatmap showing common genes that were differentially expressed (FDR < 0.05) in 3 or more cell types in the Pre‐diet versus Control (left) and Post‐diet versus Pre‐diet (right) comparisons. Genes were predominantly upregulated in Pre‐diet versus Control and downregulated in Post‐diet versus Pre‐diet, indicating reversal of transcriptional activity following ketogenic therapy. Intensity of colour reflects magnitude of log_2_ fold change (log_2_FC), with red indicating increased expression and blue indicating decreased expression. (D) Violin plot showing expression levels of *FKBP5* across major immune cell types in controls (grey), patient pre‐diet (green) and patient post‐diet (purple). *FKBP5* expression was elevated across multiple cell types pre‐diet and normalised toward control levels post‐diet, consistent with reduced stress‐response activation following KD. The most statistically significant difference in *FKBP5* expression (Post‐diet versus Pre‐diet) was observed in Neutrophils (*adj p* < 1.29 × 10^−273^), while the least significant change was seen in B cells (*adj p* = 3.35 × 10^−40^). (E) Violin plot showing expression levels of *IL1R2* across major immune cell types in control (grey), patient pre‐diet (green) and patient post‐diet (purple). *IL1R2* expression was elevated pre‐diet predominantly in Neutrophils and Monocytes, and normalised toward control levels post‐diet, indicating attenuation of inflammatory gene expression following KD. The most statistically significant difference in *IL1R2* expression (Post‐diet versus Pre‐diet) was observed in Neutrophils (*adj p* = 2.39 × 10^−242^), while the least significant change was seen in CD8^+^ T cells (*adj p* = 4.14 × 10^−11^).

In Pre‐diet versus Control comparison, total DEGs per cell type ranged from 59 to 1129. There were more downregulated DEGs than upregulated, with neutrophils and CD4^+^ T cells having the highest number of DEGs (Figure 2A). By contrast, in Post‐diet versus Pre‐diet, total DEGs per cell type ranged from 13 to 2510, and there were more upregulated DEGs than downregulated (Figure 2B). The most significant DEGs present in four or more cell types included *FKBP5*, *SMAP2, ZBTB16, IL1R2*, which were upregulated pre‐diet and downregulated post‐diet (Figure 2C). Violin plots illustrate increased expression of *FKBP5* and *IL1R2* pre‐diet, with normalisation to control levels post‐diet (Figure 2D,E; additional genes in Figure S3).

### 3.4 Enriched GO Pathways Reversed by KD in Neutrophils and CD4+ T Cells

**FIGURE 3:**
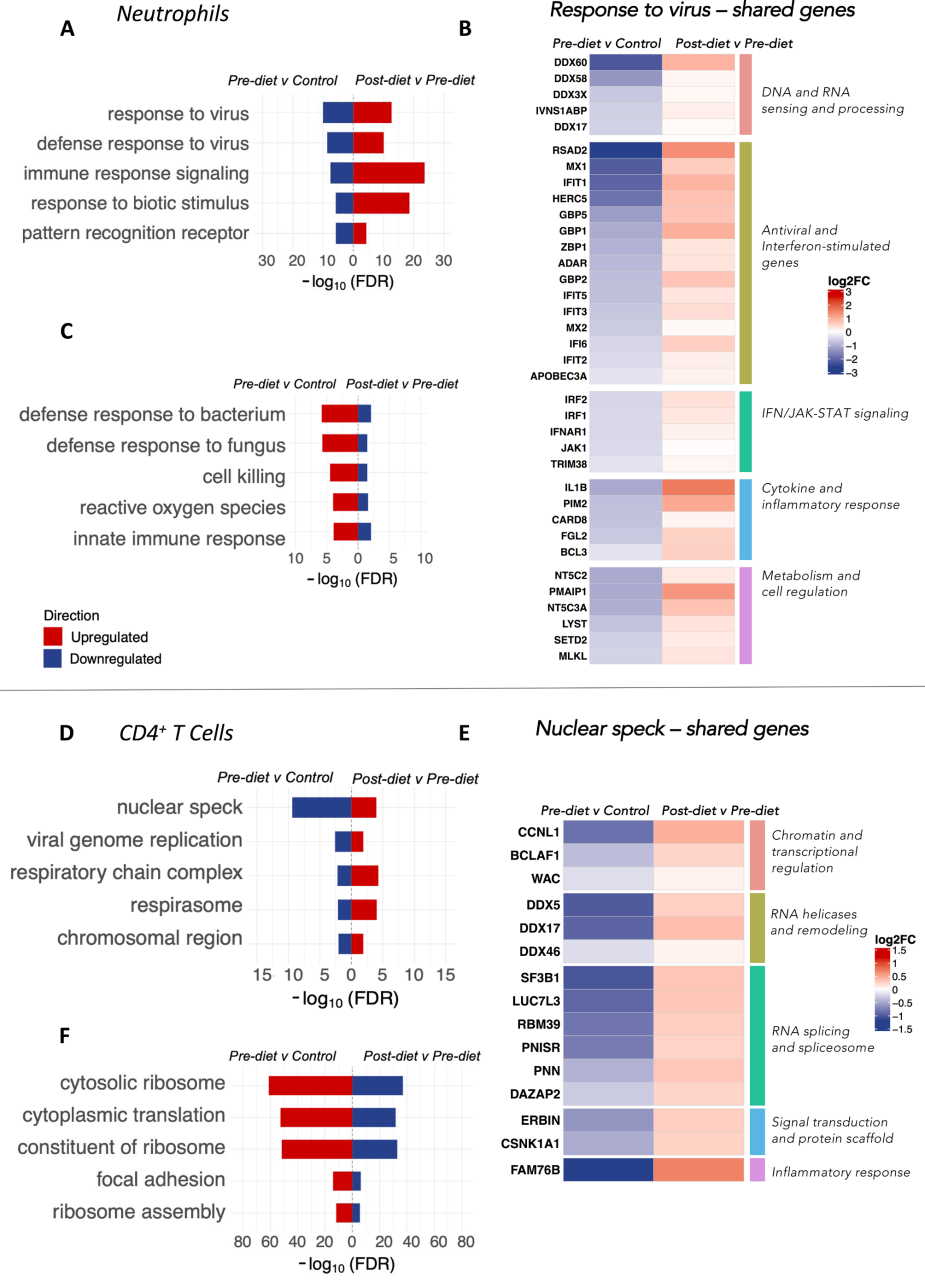
(A) Divergent barchart of Gene Ontology (GO) pathways in Neutrophils, depicting the top 5 most downregulated pathways in Pre‐diet versus Control that were subsequently upregulated in Post‐diet versus Pre‐diet. These pathways were related to immune and inflammatory (viral) processes. (B) Heatmap depicting log_2_ fold‐changes (log_2_FC) of differentially expressed genes (DEGs) in the ‘response to virus’ pathway, which was the top downregulated pathway in Neutrophils. The intensity of colour reflects the magnitude of log_2_FC. Genes downregulated in Pre‐diet versus Control are shown in blue, with darker shades indicating greater fold‐change. Genes upregulated in Post‐diet versus Pre‐diet are shown in red, with darker shades indicating greater fold‐change. (C) Divergent bar chart of GO pathways in Neutrophils, showing the top five pathways upregulated in Pre‐diet versus Control that were subsequently downregulated in Post‐diet versus Pre‐diet. These pathways were primarily related to immune and inflammatory (bacterial) processes. (D) Divergent bar chart of GO pathways in CD4^+^ T cells, showing the top five pathways downregulated in Pre‐diet versus Control that were subsequently upregulated in Post‐diet versus Pre‐diet. These pathways were associated with chromatin modification and mitochondrial function. (E) Heatmap of log_2_ fold‐changes in DEGs in the top downregulated pathway in CD4^+^ T cells—‘nuclear speck.’ The intensity of colour reflects the magnitude of log_2_FC. Genes downregulated in Pre‐diet versus Control are shown in blue, with darker shades indicating greater fold‐change, while genes upregulated in Post‐diet versus Pre‐diet are shown in red, with darker shades indicating greater fold‐change. (F) Divergent bar chart of GO pathways in CD4^+^ T cells, showing the top five pathways upregulated in Pre‐diet versus Control that were subsequently downregulated in Post‐diet versus Pre‐diet. These pathways were predominantly related to translational and ribosomal processes.

To explore targeted pathway‐level analysis of the effects of KD, neutrophils and CD4^+^ T cells were selected. In neutrophils, the most downregulated pathway in Pre‐diet versus Control that became significantly upregulated post‐diet was ‘response to virus’ (Figure 3A), driven by reversal of genes including RNA helicases (*DDX* genes), interferon‐stimulated genes (*RSAD2, IFIT* family) and interferon signalling mediators (*JAK1, IRF* genes) (Figure 3B). Conversely, the most upregulated pathway in Pre‐diet versus Control that became downregulated post‐diet was ‘defense response to bacterium’ (Figure 3C), with reversal of genes involved in pathogen recognition and inflammatory/immune signalling (Figure S3).

In CD4^+^ T cells, the most downregulated pathway in Pre‐diet versus Control comparison that became upregulated post‐diet was ‘nuclear speck’ (Figure 3D), driven by reversal of genes involved in spliceosome assembly (*SF3B1, DDX* genes, *LUC7L3*), chromatin modification (*WAC, BCLAF1*) and stress response (*BCLAF1, CSNK1A1, DAZAP2*) (Figure 3E). Conversely, the most upregulated pathway in Pre‐diet versus Control that became downregulated post‐diet was ‘cytosolic ribosome’ (Figure 3F), characterised by reversed expression of ribosomal protein genes (*RPS, RPL*) (Figure S3).

## 4 Discussion

Our analysis demonstrates the utility of scRNA‐Seq for mechanistic insights in rare disease, even at an *n* = 1 scale. Baseline *SETD1B*‐related epilepsy was characterised by broad transcriptional dysregulation, with reversal of pathways related to epigenetic, immune and stress response following KD treatment. Clinically, our patient showed substantial responsiveness to the KD, with a > 90% reduction in seizure frequency, highlighting the value of integrating multi‐omics approaches with clinical outcome measures.

At baseline, innate and adaptive immune cells in *SETD1B*‐related epilepsy exhibited dysregulation of chromatin, ribosomal, mitochondrial and inflammatory pathways. Notably, *‘*nuclear speck*’* pathways were downregulated pre‐diet but upregulated following KD therapy, suggesting beneficial effects of ketones on epigenetic regulation and RNA processing. This is mechanistically intuitive given the role of *SETD1B* in H3K4 trimethylation—an epigenetic mark associated with open chromatin and transcriptional activation. Loss‐of‐function variants in *SETD1B* reduce H3K4me3, resulting in chromatin compaction and transcriptional repression at key regulatory loci [6]. Ketone bodies produced during the KD act as HDAC inhibitors [7, 8], promoting histone modification and chromatin accessibility and may counteract the transcriptional dysregulation induced by *SETD1B* loss of function. Post‐diet upregulation of ‘nuclear speck’ supports this mechanism, indicating enhanced spliceosomal and epigenetic activity.

The KD modulated expression of key genes involved in epigenetic regulation, ribosomal proteins, stress response and immune activation in *SETD1B*‐related epilepsy. Genes associated with chromatin regulation and transcriptional control (*SETD2, CSNK1A1, BCLAF1*) were downregulated at baseline and upregulated following KD. Similarly, genes involved in RNA metabolism (*DDX* helicases) and antiviral defence (*IFIT1, IFIT2, IFIT5, IL1B* and *JAK1*) were downregulated pre‐diet and upregulated post‐diet, indicating recovery of RNA processing and interferon‐mediated antiviral pathways. These findings suggest that the KD may exert antiepileptic effects by modulating gene expression and enhancing cellular stress and immune responses [9].

By contrast, ribosomal and translational pathways were upregulated at baseline and downregulated following KD, consistent with reversal of dysregulated protein synthesis. Aberrant ribosomal protein expression has been reported in histone lysine methyltransferase disorders such as *KMT2D* Kabuki Syndrome, where ketogenic therapy reversed ribosomal dysregulation and was associated with resolution of episodes of cognitive regression [10]. The normalisation of translational activity post‐diet suggests that the KD may restore chromatin‐mediated control of protein synthesis, supporting broader recovery of transcriptional and epigenetic homeostasis in *SETD1B* deficiency.

Stress‐response mediators (*FKBP5, SMAP2, ZBTB16*) and immune signalling and activation genes (*IL6R, TLR2, TNFSF8, HLA‐E, HMGB2* and *RNASE6*) were upregulated at baseline and downregulated following KD, suggesting attenuation of a stress‐primed and inflammatory state. Notably, direct comparison of post‐diet patient samples with the age‐ and sex‐matched control showed that expression of the most upregulated genes, including *FKBP5* and *IL1R2*, decreased from markedly elevated pre‐diet levels toward gene expression observed in the control (Figure 2D,E). This pattern suggests transcriptional normalisation rather than over‐correction, consistent with a return toward a homeostatic immune and stress‐response state following KD. Such rebalancing of gene expression may support improved regulation of immune and stress‐related pathways related to clinical seizure reduction.

Limitations of this report include the examination of a single case versus control, no duplicate or longitudinal samples to assess intra‐individual variability, and scRNA‐Seq analysis of peripheral blood rather than brain cells; however, *SETD1B* is highly expressed in immune cells [11], and we propose immune cells make reasonable models of *SETD1B* dysfunction. To further explore these effects, future studies may include single‐cell proteomics and chromatin accessibility assays such as ATAC‐Seq [8] and ChIP‐Seq [12] in larger cohorts.

## 5 Conclusion

Using scRNA‐Seq, we identified cell type‐specific transcriptional dysregulation in *SETD1B*‐related epilepsy, including chromatin, immune and stress‐related pathways, many of which were reversed following KD therapy. These findings demonstrate the broad gene regulatory effects of the KD and support the utility of single‐cell transcriptomics as a precision‐medicine tool for mechanistic insight and therapeutic monitoring in rare disease.

## Author Contributions

E.T., R.C.D., V.X.H., S.P., J.P.H. conceptualised, designed and performed the experiments. E.T., A.J.A.H., M.P.M. J.A.B., R.C.D. recruited the participants. B.S.G. performed bioinformatic analysis. E.T., R.C.D., V.X.H., S.P., J.P.H., A.J.A.H., M.P.M., J.A.B., S.S.M., J.J.Y. analysed and interpreted the data. E.T., R.C.D., V.X.H. wrote the original manuscript. All authors critically revised and edited the manuscript. All authors approved the final version of the article.

## Funding

This work was supported by the National Health and Medical Research Council, 1193648.

## Ethics Statement

Ethical approval was granted by the Sydney Children's Hospitals Network Human Research Ethics Committee (HREC/18/SCHN/227, 2021/ETH00356).

## Conflicts of Interest

We confirm that we have read the Journal's position on issues involved in ethical publication and affirm that this report is consistent with those guidelines.

## Supporting information


**Figure S1:** Blood Ketone Levels over 3 months on the Ketogenic Diet.
**Figure S2:** Single‐cell RNA sequencing of SETD1B pre‐diet, post‐diet, and control.
**Figure S3:** Differentially expressed genes (DEGs) of interest.

## Data Availability

The single‐cell RNA sequencing data has been uploaded to Gene Expression Omnibus (GEO) under accession GSE319821.
