# Supplementary material for: Ketogenic Diet as an Epigenetic Therapy in SETD1B‐Related Epilepsy
Source: Ann Clin Transl Neurol. 2026 Feb 19;13(6):1268–75. doi: 10.1002/acn3.70345 (PMC13251447; doi:10.1002/acn3.70345)
Supplement: Supplementary file 1 — Figure S1: Blood Ketone Levels over 3 months on the Ketogenic Diet. Figure S2: Single‐cell RNA sequencing of SETD1B pre‐diet, post‐diet, and control. Figure S3: Differentially expressed genes (DEGs) of interest. [file ACN3-13-1268-s001.pdf]

Supplementary Methods S1

Single-cell blood RNA sequencing method

Red blood cells were depleted from whole blood by immunomagnetic negative selection, using the EasySep™ RBC depletion reagent protocol (catalog #18000, STEMCELL Technologies, Vancouver, Canada), preserving all leukocyte populations, including granulocytes. Three HIVE devices (CLX version, Honeycomb Biotechnologies, Inc., USA) (2 samples from patient and 1 control) were each loaded with approximately 30,000 cells in 1 mL of DPBS + 1% FBS, followed by 3 mL of cell medium (DPBS + 1% FBS). Leukocytes were loaded into the HIVE system within an hour of blood sampling from all individuals to minimise *ex vivo* neutrophil activation. Single cells settled into picowells within the HIVE devices, which contained 3' transcript-capture beads. HIVEs were centrifuged at 30 x g for 3 min to ensure cell settling. Following media removal, HIVEs were washed twice with 2 mL of sample wash solution. After removing the wash solution, 2 mL of cell preservation solution was added, and the cell-loaded HIVEs were frozen at -80 °C (Honeycomb Biotechnologies, Inc. USA Sample Capture protocol).

Frozen devices were transported to the Australian Genome Research Facility Ltd (AGRF Ltd, Westmead, Australia) for single-cell next-generation sequencing (NGS) library preparation, according to the manufacturer's protocol (Honeycomb Biotechnologies, Inc. USA Sample Capture protocol). HIVE devices were sealed with a semi-permeable membrane, enabling on-device lysis and hybridisation. Beads with captured transcripts were extracted from the HIVE device by centrifugation, and subsequent library preparation steps were performed in a 96-well plate format. Library size distribution and quality were assessed using a TapeStation 2200 platform with a D5000 ScreenTape System (Agilent Technologies, Santa Clara, CA, USA). The concentration of final pooled libraries was determined by qPCR. Final HIVE scRNA-seq libraries were sequenced on an Illumina NovaSeq X sequencer (AGRF Ltd, Melbourne, Australia) using kit-specific primers.

Single-cell RNA sequencing bioinformatic analysis

Cells with a high mitochondrial transcript ratio (>0.15) were excluded. Normalisation was performed using *SCTransform* function in the *Seurat* R package (v5.3.1). Immune cell identities were assigned using *scType* and *scPred (v.1.9.2).* Merged datasets were split by cell type, and separately normalised, scaled, and integrated across patients using *harmony (v1.2.4).* Uniform manifold approximation and projection (UMAP) embeddings were generated from the first 30 principal components. Differentially expressed genes were identified using the *FindMarkers* function in *Seurat* with a false discovery rate (FDR) <0.05.

Pathway Enrichment Analysis Using ORA

All analyses were performed using R (v4.5.2). Gene Ontology (GO) over-representation analysis (ORA) was conducted on DEGs from Pre-diet vs Control and Post-diet vs Pre-diet comparisons using the *enrichGO()* function from the *clusterProfiler* package (v.4.19.1) with *OrgDb = org.Hs.eg.db*. Separate enrichment analyses were performed for upregulated and downregulated gene sets. Redundant GO terms were reduced using the *simplify()* function with a similarity cutoff of 0.7. Enrichment results were visualised using *ggplot2* (v4.0.0). To explore baseline SETD1B gene expression, a dot plot was generated for the top 10 significantly enriched pathways per direction, filtered to include GO terms shared across ≥2 immune cell types in Pre-diet vs Control (Figure 1C). Terms were grouped by functional category (e.g., translation, immune, mitochondrial), and shaded background bands were added to visually distinguish categories. Dot size reflected $-\log_{10}$(FDR), and colour indicated directionality (up- or downregulated). Supporting packages included *dplyr* (v1.1.4), *forcats* (v1.0.1), and *stringr* (v1.5.2). To assess pathway changes induced by diet between groups, divergent bar charts were generated for the top 5 pathways in Pre-diet vs Control that reversed direction in expression in Post-diet vs Pre-diet in Neutrophils and $CD4^+$ T cells (Figure 3).

Gene-Level Expression Analysis

Gene-level expression changes were visualised using heatmaps and violin plots. A heatmap displaying $\log_2$ fold-change values was generated for genes differentially expressed in ≥3 immune cell types, across Pre-diet vs Control and Post-diet vs Pre-diet. Genes were ordered by number of cell types expressed and average $\log_2$FC and visualised in *ggplot2* with symmetric colour scaling and fixed axis order.

To highlight key stress and immune-related genes expressed across ≥4 cell types (*FKBP5*, *IL1R2*, *SMAP2*, *ZBTB16*), violin plots were generated to compare expression in Pre-diet vs Control and Post-diet vs Pre-diet. Normalised expression values were extracted from the SCT assay of the processed *Seurat* object, and violin plots were rendered using *ggplot2*.

Targeted expression heatmaps were generated using the *ComplexHeatmap* package (v2.26.0) for genes shared among top enriched pathways, including 'response to bacterium' and 'response to virus' (Neutrophils), and 'cytosolic ribosome' and 'nuclear speck' ($CD4^+$ T cells). Gene × comparison matrices were formatted using *tidyverse* tools.

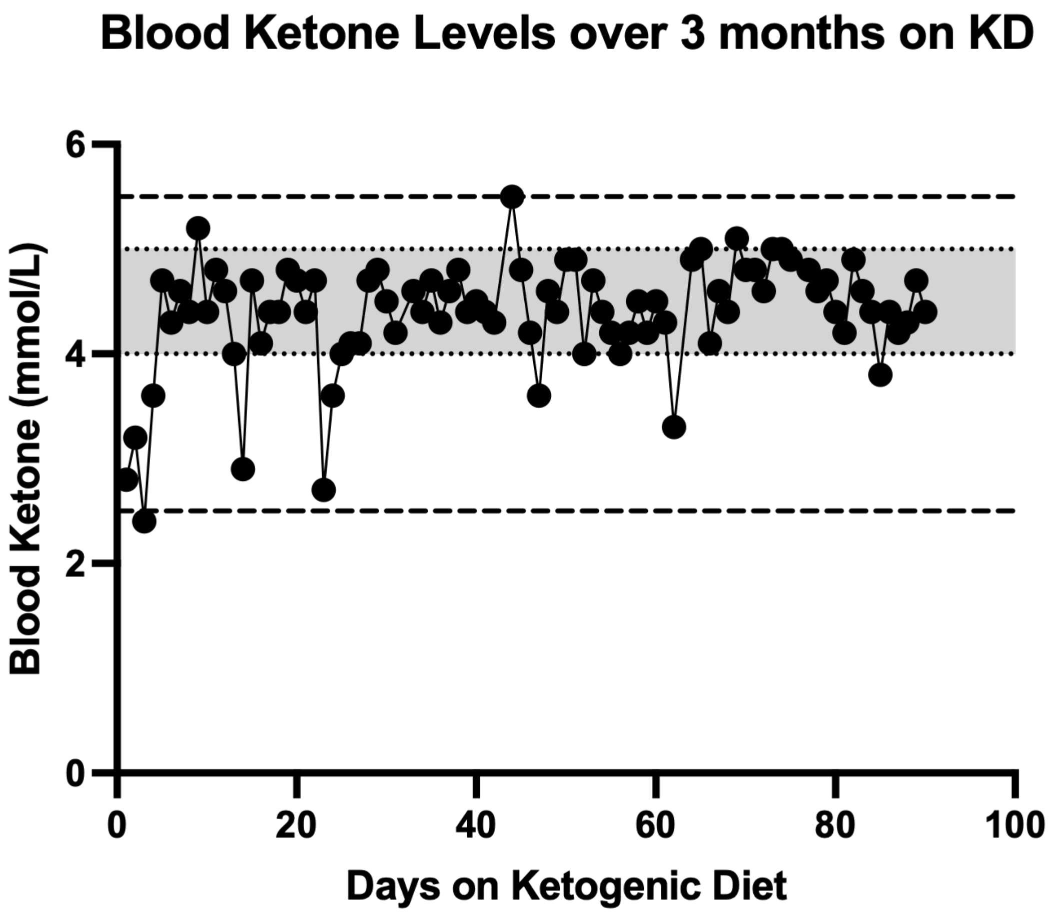

Supplementary Figure S1: Blood Ketone Levels over 3 months on the Ketogenic Diet. Measures were taken twice-daily (morning and evening) and the highest level plotted. The dashed lines indicate ideal blood ketone range for epilepsy (2.5-5.5mmol/L). The grey area indicates the reported blood ketone range (4-5mmol/L) whereby seizures were controlled ay 0-3 per day. Blood ketones remained in ideal range over the course of 3 months on KD, supporting good diet compliance.

A

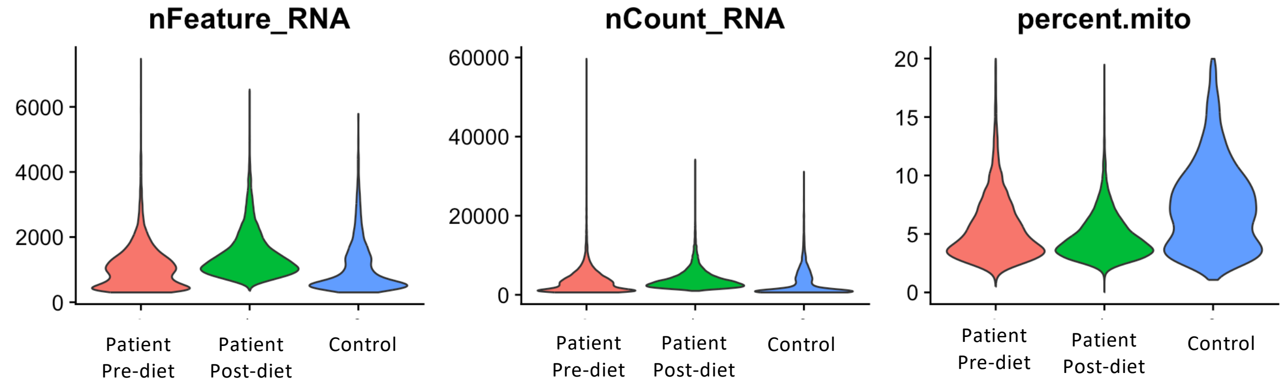

B

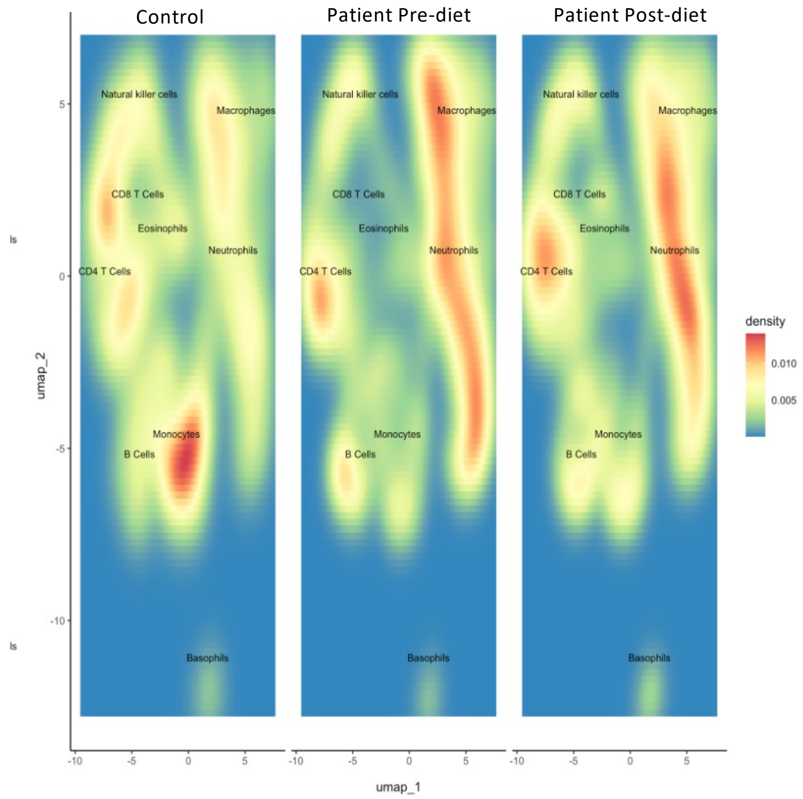

Supplementary Figure S2: Single-cell RNA sequencing of SETD1B pre-diet, post-diet and control

A) Number of features, counts and mitochondrial percentage across samples

B) UMAP density plot of cell types across samples. The colour gradient (green to red) represents cell density, with red indicating cell types with higher cellular density and green indicating cell types with lower cellular density.

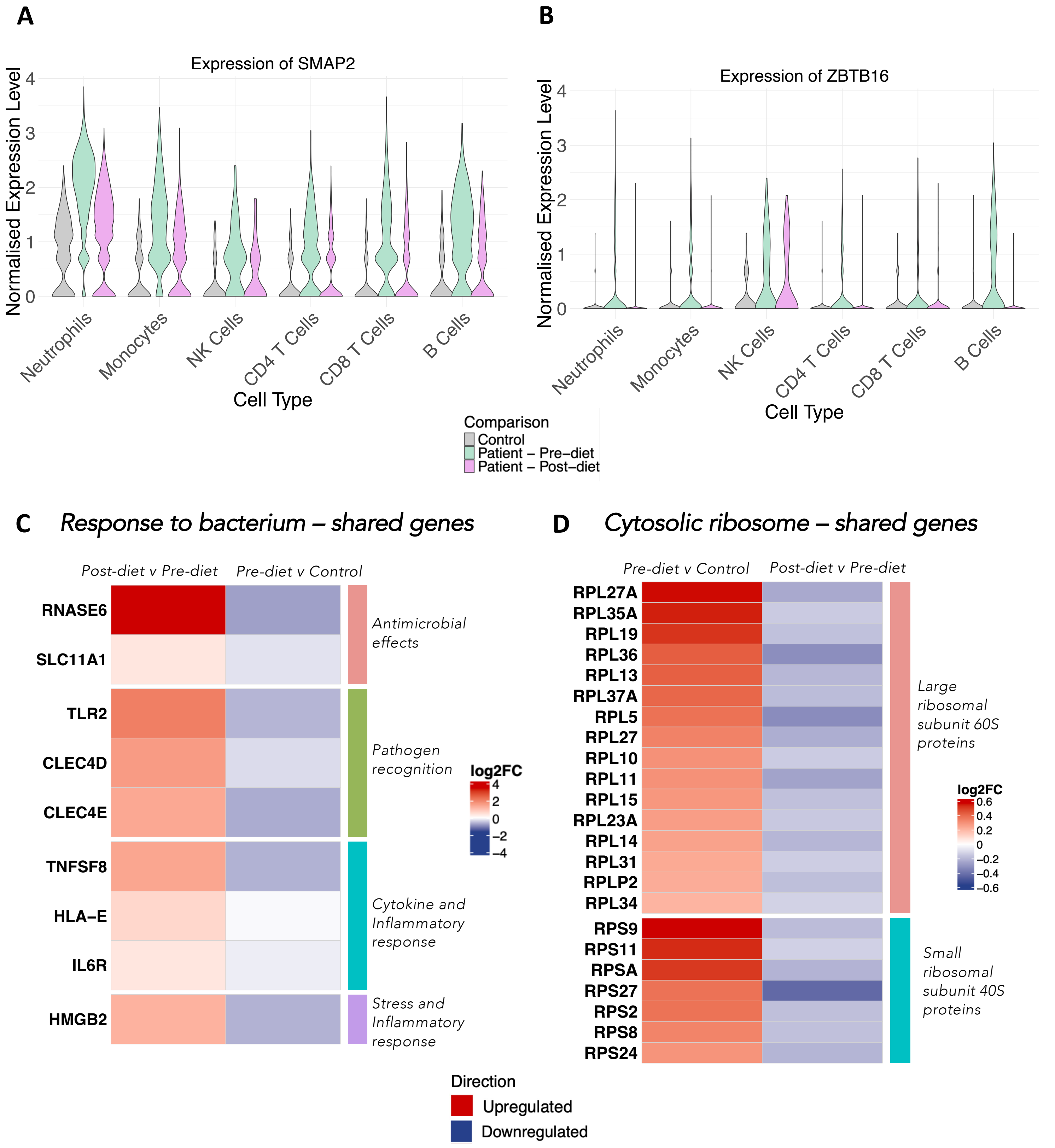

Supplementary Figure S3: Differentially expressed genes (DEGs) of interest – KD in SETD1B-related absence epilepsy

A) Violin plot of SMAP2 expression across patient pre-diet, post-diet, and control

B) Violin plot of ZBTB16 expression across patient pre-diet, post-diet, and control

C) Heatmap of $\log_2$ fold-changes in DEGs in the top upregulated pathway in Neutrophils - ‘response to bacterium.’

D) Heatmap of $\log_2$ fold-changes in DEGs in the top upregulated pathway in CD4+ T Cells - ‘cytosolic ribosome.’
